# Action against birth defects: if not now, when?

**DOI:** 10.1080/16549716.2024.2354002

**Published:** 2024-05-31

**Authors:** Kathleen Strong, Judith Robb-McCord, Salimah Walani, Cecilia Mellado, Lorenzo D. Botto, Guillermo Lay-Son, Theresa Diaz, Tahmina Banu, Kokila Lakhoo, Anshu Banerjee

**Affiliations:** a Department of Maternal, Newborn, Child and Adolescent Health and Aging Department, World Health Organization, Geneva, Switzerland; b Independent (formerly March of Dimes), Chapel Hill, NC, USA; c MiracleFeet, Scottsdale, AZ, USA; d School of Medicine, Pontificia Universidad Católica de Chile, Santiago, Chile; e Dept. of Genetics, University of Utah, Salt Lake City, UT, USA; f Chittagong Research Institute for Children’s Surgery, Dhaka, Bangladesh; g University of Oxford and Oxford University Hospitals, Oxford, UK

**Keywords:** Child mortality, newborn mortality, birth defects, disability, sustainable development goals

## Abstract

**Background:**

More children are surviving through interventions to address the infectious causes of under-5 mortality; subsequently, the proportion of deaths caused by birth defects is increasing. Prevention, diagnosis, treatment and care interventions for birth defects are available but are needed where the burden is highest, low-and-middle-income countries.

**Objectives:**

A selection of birth defect focused publications, conferences, and World Health Assembly resolutions from 2000 to 2017 show that global efforts were made to raise the profile of birth defects in global public health. However, recent donor support and national government interest has waned. Without concerted global action to improve primary prevention and care for children born with birth defects, the Sustainable Development Goal targets for child survival will not be met.

**Results:**

Birth defects make up 8% and 10% of global under-5 and neonatal deaths respectively, making them significant contributors to preventable loss of life for children. Survivors face long-term morbidity and lifelong disability which compounds the health and economic woes of individuals, families, communities and society as a whole. Demographic changes in sub-Saharan Africa portend a growing number of births with 1.6 billion projected from 2021 to 2050. More births and better survival without effective prevention and treatment for birth defects translates into more mortality and disability from birth defects.

**Conclusions:**

We recommend interventions for prevention of birth defects. These are evidenced-based and affordable, but require low- and middle-income countries to strengthened their health systems. Action against birth defects now will prevent premature deaths and long-term disability, and lead to stronger, more resilient health systems.

## Background

An estimated 8 million newborns are born with a birth defect each year, and of these an estimated 240,000 die worldwide in their first month of life, making birth defects a leading cause of both neonatal and postneonatal child deaths [1]. In addition, some of the 1.9 million babies stillborn in 2021 will be the result of a congenital anomaly [2]. Those who survive a serious birth defect often face a lifetime of severe disability and stigma.

**Figure 1.**
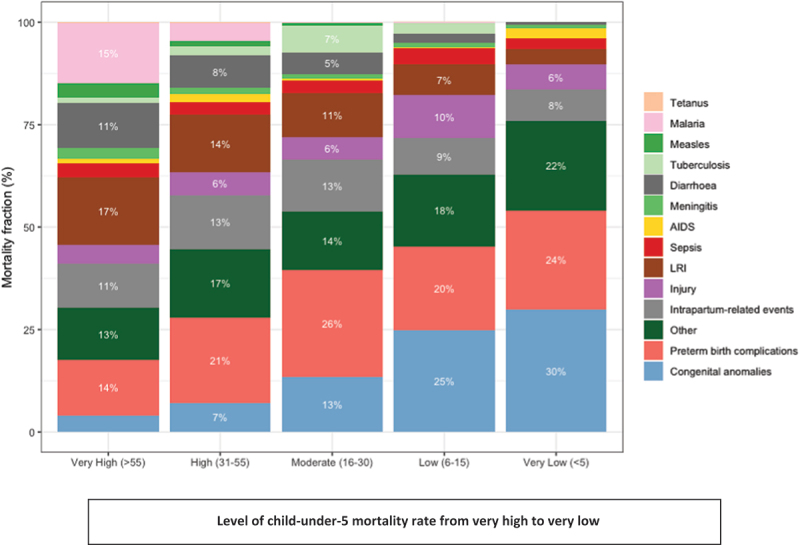
Proportional mortality for children under-5 years according to all-cause mortality rate categories (very high, U5MR greater than 55 deaths per 1000 live births to very low, U5MR less than 5 deaths per 1000 live births). Cause specific mortality is from the child and adolescent cause of death estimation group, 2021(1). Congenital anomalies increase as a proportion of total under-5 mortality as survival improves.

The leading causes of birth defects globally are congenital heart anomalies and neural tube defects [3]. These two preventable and/or treatable conditions account for over half of all neonatal deaths due to birth defects [3]. Crucially, as child survival improves through improved control of infectious childhood diseases, birth defects account for an increasing proportion of under-5 deaths, up to 30% in low-mortality settings [1,4] (Figure 1).

The vast majority of newborns with a birth defect, including an estimated 94% of those with severe disorders, are born in low- and middle-income countries (LMICs) where diagnostic, treatment and management services are limited [3]. Absence of birth defects surveillance programs in most LMICs coupled with a lack of global consensus on birth defect terminology, means that we vastly underestimate the true burden of birth defects in these countries [5].

The ability to identify, treat and successfully manage birth defects is key to building bridges from survival to optimal growth and development for affected children and to eliminate discrimination, unequal educational opportunities, physical and psychological violence [6]. Newborns and children with birth defects are often unable to receive the treatment and care they need due to delayed diagnoses, lack of referral systems, limited access to facilities and resources, shortage of trained healthcare providers, misinformation and misconceptions, all of which are compounded by stigma and discrimination [6]. In brief, for many birth defects the tools for primary prevention and effective management are well known and tested [7]: much of the current burden of birth is a failure of implementation. We argue that immediate action from the global public health community is needed to fund effective primary prevention and early detection (e.g. via expanded newborn screening), and improved early diagnostic services. These actions, coupled with effective, family-centered medical and surgical treatment and follow up that supports early childhood development will support optimal growth and development for all affected children.

## Sustainable development goal child survival targets will not be met by 2030 without action against birth defects

National governments and the global public health community are investing in interventions to accelerate progress towards achieving the Sustainable Development Goals (SDGs) for child survival,^1^^1^SDG 3.2.1 to reduce under-5 mortality to at least as low as 25 deaths per 1000 live births and SDG 3.2.2 to reduce neonatal mortality to at least as low as 12 deaths per 1000 live births. through the Global Strategy for women’s, children’s and adolescents’ health [8,9], Ending Preventable Maternal Mortality [10] the Every Newborn Action Plan (ENAP) [11], and the Child Survival Action initiative [12]. These global initiatives are important accountability mechanisms for child survival and development; yet birth defects are not specifically addressed in any of them. Surveillance, prevention and providing care for children with birth defects are important strategies to reach the SDG child survival targets and align with the SDG commitment to equity to “leave no one behind” [8].

Unfortunately, despite World Health Assembly Resolutions, a global report, and multiple calls for action by professional and patient organizations (Table 1), little progress has been made in championing birth defect prevention and care. Lack of progress is especially concerning as the impact of birth defects is greatest where need for progress is largest. The highest rates of both stillbirths and child deaths occur in sub-Saharan Africa, a region that will see 339 million births and a 12% increase in the under-5 population, which will reach more than 200 million by 2030 [13]. This demographic reality coupled with the fact that the majority of those born with severe disorders are in low-and-middle income countries, especially in sub-Saharan Africa, makes the need for equal access for prevention, screening and curative treatment an urgent priority for global public health.

**Table 1.** Highlights of global reports, conferences, professional association statements and UN resolutions. Many other national advocacy actions have been produced in the last two decades. Collectively, very little result has come from these efforts.

Organization	Action	Date	Result
March of Dimes	International Conference on Birth Defects and Disabilities in the Developing World (ICBD)	2001, 2005, 2007, 2009, 2011, 2013, 2015, 2017, 2020, 2023	Number of conferences sponsored until the final conference in 2023 in Santiago Chile; No further conferences will be supported by March of Dimes.
March of Dimes, World Health Organization and other partners	Global Report on Birth Defects^2^	2006	Global report on burden of birth defects, showing the heavy burden on low and middle income countries
World Health Organization	World Health Assembly 63 passed resolution 63.17 recognized the importance of birth defects as a cause of stillbirths and neonatal mortality^3^	2010	WHO Member States agreed to promote primary prevention, screening, surveillance, and to improve the health of children born with birth defects
International Conference on Birth Defects and Disabilities in the Developing World (ICBD)	Consensus Statement from professionals working in birth defects^4^	2015	Called for actions to ‘maximize the opportunity for every woman and couple to have a healthy child and to reduce the mortality and severe disability associated with potentially avoidable congenital disorders and their consequences’
International Conference on Birth Defects and Disabilities in the Developing World (ICBD)	Consensus Statement from professionals working in birth defects^5^	2017	Called for improved screening, detection, treatment, care and research into birth defects
World Health Organization	World Health Assembly 67 passed resolution 76.19 to accelerate efforts to prevent micronutrient deficiencies through safe and effective food fortification, a population-wide health intervention to prevent some severe birth defects of the brain and spine.^6^	2023	WHO urges Member States to implement large scale food fortification as an evidence-based and cost-effective intervention to fight the consequences of vitamin and mineral deficiencies, including spina bifida and other neural tube defects

## Effective interventions to prevent, treat and care for children with birth defects exist and their use requires investment from health systems

Evidence shows that there are affordable interventions available now to prevent many cases of birth defects. Examples include vaccination against rubella, universal salt iodization and food fortification with folic acid [7]. The latter is a clear example of an population-based and relatively inexpensive intervention that can reduce the impact of birth defects by reducing the number of children born with a neural tube defect [14]. At the same time, health systems – particularly those in LMICs – need to invest in the development of comprehensive policies and approaches to ensure newborns and children with birth defects have universal access to affordable, timely and appropriate medical, surgical and long-term developmental care without financial hardships [15]. Health systems need to have measures in place to protect families and caregivers from the devastating impact of high out-of-pocket expenditures related to the care their child will need. As with other health services, treatment and care for children with birth defects and disabilities needs to be respectful, nurturing and based on quality-of-care principles [16]. Families need to have voice in the care their children receive, and children need to be protected from violence and discrimination to survive and thrive as productive, valued members of society. We make the following recommendations to support prevention and treatment of birth defects and to support care for people living with them, cognizant that sectors other than health will also need to be strengthened:^2^^2^Christianson A, Howson C, Modell B. March of Dimes: Global.Report on Birth Defects, the Hidden Toll of Dying and Disabled Children. New York: March of Dimes Birth Defects Foundation; 2006.^3^^3^World Health Organization. Sixty-Third World Health Assembly.Resolution 63.17. Birth Defects. Geneva: World Health Organization; 2010.^4^^4^Darmstadt GL, Howson CP, Walraven G, Armstrong RW, Blencowe HK, Christianson AL, et al. Prevention of Congenital Disorders and Care of Affected Children: A Consensus Statement. JAMA Pediatr. 2016;170(8):790–793. do i:1 0.1001/jamapediatrics.2016.0388.^5^^5^Zarante I, Hurtado-Villa P, Walani SR, Kancherla V, López Camelo J, Giugliani R, et al. A consensus statement on birth defects surveillance, prevention, and care in Latin America and the Caribbean. Rev Panam Salud Publica. 2019;43:e2. https://doi.org/10.26633/RPSP.2019.2^6^^6^World Health Organization. Sixty-Seventh World Health Assembly.Resolution 76.19. Accelerate efforts to prevent micronutrient deficiencies through safe and effective food fortification, a population-wide health intervention to prevent some severe birth defects of the brain and spine. Geneva: World Health Organization; 2023. https://apps.who.int/gb/ebwha/pdf_files/WHA76/A76_R19-en.pdf

(1) Expand and integrate birth defects prevention into clinical and public health practice.
  - Introduce and scale evidence-based risk reduction interventions into national health campaigns, health and wellness programs, preconception and antenatal care [17].
  - Renew the focus on effective primary prevention, including but not limited to population-based food fortification with folate and iodine; integration of rubella vaccination into the Expanded Programme on Immunization; improved nutrition for girls and women including micronutrient supplementation before and during pregnancy; family planning knowledge and services; management of chronic illness and infection from before conception through prenatal care; and reducing/eliminating exposure to harmful substances, drugs and environmental pollutants prior to and during pregnancy [18].
(2) Improve diagnosis, management and care within health care systems.
  - Strengthen and expand newborn screening, early detection, and diagnosis of birth defects, followed by referral to appropriate medical and surgical treatment that is family-centered and includes early childhood development and follow-up care [19].
  - Promote a multidisciplinary approach for specialized care such as surgery, mental health, and speech and occupational therapy, among other professional services.
  - Train health professionals to practice respectful and family-centered care.
  - Integrate prevention, identification, diagnosis and care of newborns and children with birth defects into universal health coverage and reproductive, maternal, newborn, child and adolescent health policies, guidelines, strategies, and action plans at the national, regional, and sub-regional levels.
(3) Protect the rights of individuals affected by birth defects.
  - Promote a rights-based and equitable approach to diagnosis and care (including respectful and family-centered care) in newborns and children, across their lifespan.
  - Listen to, empower, and incorporate parent and family voices into all policies, strategies and practices targeting birth defects.
  - Ensure timely access to screening, diagnosis, and care, including surgical care, for newborns and children with birth defects as an essential aspect of health coverage.
  - Eliminate violence, stigma, and discrimination against persons with birth defects.
(4) Support and expand sustainable information systems to generate key data on birth defects – their occurrence, risk factors, and outcomes – throughout the lifespan.
  - Develop and advocate for standard definitions for birth defects [5].
  - Establish new estimates for the global burden of birth defects.
  - Strengthen registration of births and of children born with birth defects.
  - Expand public health surveillance systems to monitor birth defects occurrence, measure risk factors, and monitor health outcomes with follow-up that extends beyond the first year of life [20].
  - Link surveillance to birth defects treatment and care by creating referral pathways.
(5) Dedicate funds and resources at the global and national level to support a comprehensive approach to the prevention, surveillance, screening, diagnosis and treatment of newborns and children with birth defects [21].

## Actions needed now and into the future

While we applaud the reductions in neonatal and child mortality globally, we know that as the overall childhood mortality rates decline, birth defects become a larger proportion of the causes of neonatal and child under-5 deaths. To further reduce neonatal and under-5 mortality, particularly in LMICs struggling to meet SDG targets for newborn and child survival, global efforts to prevent birth defects and to care for affected children need to be expanded and reinforced now.

Our recommendations align with the core principles of Universal Health Coverage (UHC) and the broader Reproductive, Maternal, Newborn, Child and Adolescent Health agenda and must be placed within UHC commitments and with renewed energy around primary health care. Prevention, surveillance, screening, diagnosis, treatment and care for newborns and children with birth defects are important strategies to achieve established SDG and ENAP targets. In the absence of a significant global response, further gains in child survival will be undermined by preventable mortality due to birth defects and we will not meet the neonatal and under-5 SDG survival targets.

Children with birth defects are subjected to multiple inequities – they have functional limitations, poorer health, and shorter life spans. We call for immediate action from the global public health community to fund effective three crucial areas for meaningful progress: a) primary prevention; b) early diagnosis, including newborn screening and early detection of birth defects; and c) appropriate medical and surgical treatment that is family-centered and includes follow-up and support for early childhood development. Actions in these three priority areas will be effective to the extent that they are included in comprehensive and sustained maternal, newborn and child health frameworks, action plans, and funding priorities – global as well as national. Through such sustained action, we can fulfill the SDG commitment to leave no one behind and ensure brighter futures for millions of children born with birth defects and disabilities.

## Supplementary Material

title_page_GHA.docx

## Paper context

- As child survival continues to improve through concerted efforts against infectious childhood diseases, the proportion of under-5 mortality due to birth defects is increasing.
- We present recommendations for effective primary prevention, expanded newborn screening, early detection and diagnosis of birth defects, followed by referral to appropriate medical and surgical treatment that are needed to prevent deaths and life-long disability and continue progress toward Sustainable Development Goal child survival targets for 2030.
- We argue that surveillance, prevention, treatment and providing care for children with birth defects are important strategies to reach the Sustainable Development Goal child survival targets and align with the commitment to equity of the Sustainable Development Goals, ‘to leave no one behind’.
