# Supplementary material for: Action against birth defects: if not now, when?
Source: Glob Health Action. 2024 May 31;17(1):2354002. doi: 10.1080/16549716.2024.2354002 (PMC11146259; doi:10.1080/16549716.2024.2354002)
Supplement: title_page_GHA.docx [file ZGHA_A_2354002_SM8825.docx]

**Action against birth defects: if not now, when?**

Kathleen Strong^1^, Judith Robb-McCord^2^, Salimah Walani^3^, Cecilia Mellado^4^, Lorenzo D. Botto^5^, Guillermo Lay-Son^6^, Theresa Diaz^7^, Tahmina Banu^8^, Kokila Lakhoo^9^, Anshu Banerjee^10^

(First two authors joint co-authors)

Corresponding Author: Kathleen Strong, Department of Maternal, Newborn, Child and Adolescent Health and Aging, World Health Organization, Geneva Switzerland. Email: [strongk@who.int](mailto:strongk@who.int)

Department of Maternal, Newborn, Child and Adolescent Health and Aging Department, World Health Organization, Geneva Switzerland. Email: [strongk@who.int](mailto:strongk@who.int)

^2^Independent (formerly March of Dimes), Chapel Hill North Carolina USA. Email: [judithrobbmccord@gmail.com](mailto:judithrobbmccord@gmail.com)

^3^ MiracleFeet USA. Email: [walani@post.harvard.edu](mailto:walani@post.harvard.edu)

^4^ School of Medicine, Pontificia Universidad Católica de Chile, Santiago, Chile. Email: [cmellado@med.puc.cl](mailto:cmellado@med.puc.cl)

^5^ Dept. of Genetics, University of Utah, Salt Lake City USA. Email: [Lorenzo.Botto@hsc.utah.edu](mailto:Lorenzo.Botto@hsc.utah.edu)

^6^ School of Medicine, Pontificia Universidad Católica de Chile, Santiago, Chile. Email: [grlayson@med.puc.cl](mailto:grlayson@med.puc.cl)

^7^ Department of Maternal, Newborn, Child and Adolescent Health and Aging Department, World Health Organization, Geneva Switzerland. Email: [tdiaz@who.int](mailto:tdiaz@who.int)

^8^ Chittagong Research Institute for Children’s Surgery, Dhaka, Bangladesh. Email: [proftahmina@gmail.com](mailto:proftahmina@gmail.com)

^9^ University of Oxford and Oxford University Hospitals, Oxford UK. Email: [kokila.lakhoo@paediatrics.ox.ac.uk](mailto:kokila.lakhoo@paediatrics.ox.ac.uk)

^10^ Department of Maternal, Newborn, Child and Adolescent Health and Aging Department, World Health Organization, Geneva Switzerland. Email: [banerjeea@who.int](mailto:banerjeea@who.int)

**Word count:** 1,667, excluding abstract (250)

**Abstract:**

*Background:* More children are surviving through interventions to address the infectious causes of under-5 mortality; subsequently, the proportion of deaths caused by birth defects is increasing. Prevention, diagnosis, treatment and care interventions for birth defects are available but are needed where the burden is highest, low-and-middle-income countries.

*Objectives:* A selection of birth defect focused publications, conferences, and World Health Assembly resolutions from 2000 to 2017 show that global efforts were made to raise the profile of birth defects in global public health. However, recent donor support and national government interest has waned. Without concerted global action to improve primary prevention and care for children born with birth defects, the Sustainable Development Goal targets for child survival will not be met.

*Results:* Birth defects make up 8% and 10% of global under-5 and neonatal deaths respectively, making them significant contributors to preventable loss of life for children. Survivors face long-term morbidity and lifelong disability which compounds the health and economic woes of individuals, families, communities and society as a whole. Demographic changes in sub-Saharan Africa portend a growing number of births with 1.6 billion projected from 2021 to 2050. More births and better survival without effective prevention and treatment for birth defects translates into more mortality and disability from birth defects.

*Conclusions:* We recommend interventions for prevention of birth defects. These are evidenced-based and affordable, but require low-and middle-income countries to strengthened their health systems. Action against birth defects now will prevent premature deaths and long-term disability, and lead to stronger, more resilient health systems.

**Key words:** Child mortality, Newborn mortality, Birth Defects, Disability, Sustainable Development Goals
